# Vision-Enabled AI scribes reduce omissions in clinical conversations: evidence from simulated medication histories

**DOI:** 10.1038/s41746-026-02494-9

**Published:** 2026-02-26

**Authors:** Bradley D. Menz, Nicholas L. Scarfo, Natansh D. Modi, Erik Cornelisse, Lee X. Li, Jin Quan Eugene Tan, Jimit Gandhi, Dorsa Maher, Dib Kousa, Kezia Daniel, Vidya Menon, Stephen Bacchi, Ross A. McKinnon, Michael D. Wiese, Andrew Rowland, Michael J. Sorich, Ashley M. Hopkins

**Affiliations:** 1 College of Medicine and Public Health, Flinders Health and Medical Research Institute, Flinders University, Adelaide, South Australia; 2 SA Pharmacy, Southern Adelaide Local Health Network, Adelaide, South Australia; 3 Clinical and Health Sciences, Adelaide, University of South Australia, Adelaide, South Australia; 4 SA Pharmacy, Central Adelaide Local Health Network, Adelaide, South Australia; 5 Department of Neurology, Northern Adelaide Local Health Network, Adelaide, South Australia

**Keywords:** Health care, Mathematics and computing, Medical research

## Abstract

Most ambient AI medical scribes process audio only, omitting clinically important visual details. We developed a vision-enabled AI scribe using Google’s Gemini model and Ray-Ban Meta smart glasses to document medication histories—a task requiring both audio and visual input. Ten clinical pharmacists video-recorded 110 simulated medication history interviews. Following iterative prompt engineering on 10 training recordings, the scribe was evaluated on 100 test recordings (2160 data points) across patient details and medication-specific fields. The vision-enabled scribe achieved 98% overall accuracy (2114/2,160 data points), ranging from 96% for patient details to 99% for dosing directions and indication. Video input significantly outperformed audio-only processing (98% vs 81%, *P* < 0.001), primarily through reduced omissions (10 vs 358 errors). Vision-enabled AI scribes substantially improved documentation accuracy for tasks requiring visual input, demonstrating potential to markedly reduce omission errors in clinical documentation.

## Introduction

The integration of generative artificial intelligence (AI) into clinical workflows is advancing rapidly^1–6^. Among the earliest and most widely adopted applications are ambient AI medical scribes, which use audio recorded during clinician–patient conversations to help automatically generate structured clinical documentation^1,7–11^. These tools promise to substantially reduce administrative burden, standardise documentation and enhance both clinician efficiency and the time available for patient engagement^10,12–16^. However, because current AI medical scribes process only audio input, this limits their ability to capture the whole clinical context^17,18^. In real-world consultations, essential health information is often multimodal, encompassing both visual inspection of physical items, including medication containers, labels, devices, and nonverbal information conveyed by the patient’s appearance, posture, and behaviour^6,19–21^. Audio-only scribes are therefore vulnerable to omission, inconsistency or misinterpretation when details are visible but unspoken. Medication-history conversations exemplify this challenge, as they combine visible artefacts (such as packaging, labels, and visual cues) with spoken information from clinicians and patients. Some details will inherently be visual, while others can be conveyed through both visual and spoken means. This duplication can be advantageous for AI, as it allows for immediate cross-checks that can help minimise omissions and transcription errors.

Recent advances in multimodal generative AI now allow models to process both audio and visual inputs simultaneously^6,19,22^. This capability creates the potential for AI medical scribes that are more context-aware, perceiving not only what is said but also what is seen. Such functionality could overcome the limitations of audio-only tools and significantly improve performance in documenting many clinical tasks. In this study, we developed and evaluated a vision-enabled AI scribe built on Google’s Gemini model. The AI scribe was evaluated for the accuracy of scribing medication histories from simulated pharmacist-patient interviews that were video recorded with Ray-Ban Meta smart glasses. The secondary aim was to compare the AI scribe’s accuracy when processing the video (i.e. visuals + audio) input compared to the audio-only input.

## Results

### Training Set Performance

Across the 10 training-simulated medication history video recordings, the AI scribe was trained on 30 patient detail data points (i.e. ten data points for each of patient name, date of birth, and medication allergies across the 10 recordings) and 180 medication-specific data points (i.e. 36 data points for each of medication name, strength and form, dosing directions, indication, and relevant clinical notes across the 10 recordings). This yielded a total of 210 data points within the training set. Following iterative prompt engineering, the AI scribe correctly scribed 99% (208/210) of data points in the training data. The supplementary information presents the final prompts underpinning the engineered AI medication history scribe.

### Accuracy in the Test Set

Across the 100 test simulated medication history video recordings, the AI scribe was evaluated on 300 patient detail data points (i.e. 100 data points for each of patient name, date of birth, and medication allergies across the 100 recordings) and 1860 medication-specific data points (i.e. 372 data points for each of medication name, strength and form, dosing directions, indication, and the clinical notes field across the 100 recordings). This yielded a total of 2160 data points evaluated within the test set. With regards to establishing the pharmacist scribed references in the test set, when reviewed by the second pharmacist there were 45 data fields flagged for review by the third reviewer; of these only 11 required updating—in part, representing differences that can occur due to clinical subjectivity.

**Fig. 1:**
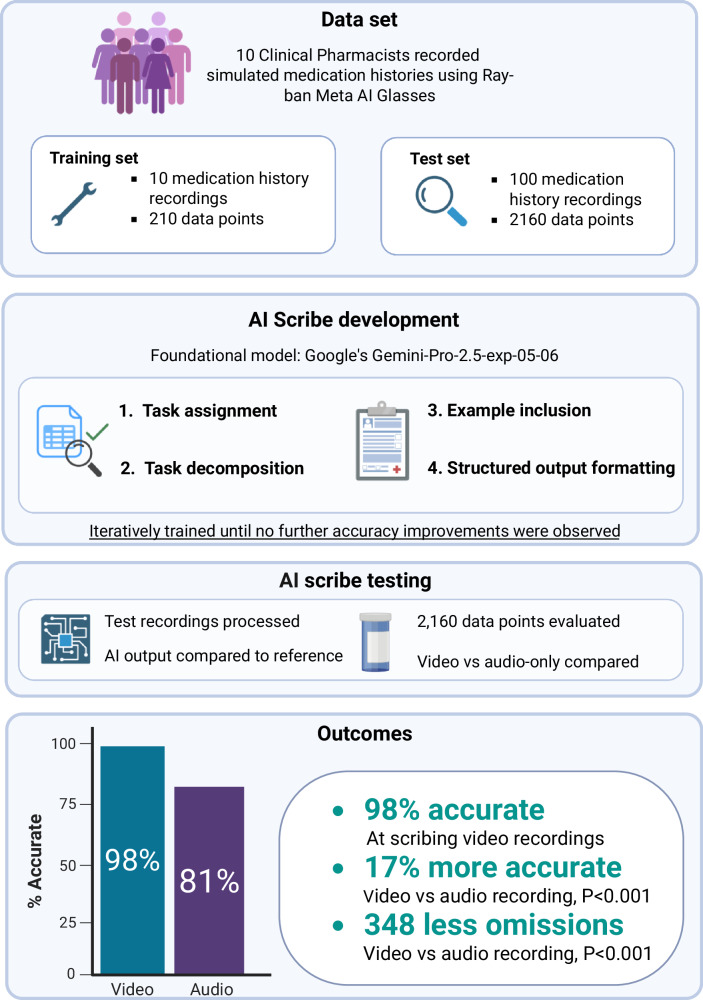
Overview of study dataset, development, evaluation, and key results. Created in BioRender. Rowland, A. (2025) https:// BioRender.com/9ennk4n.

**Fig. 2:**
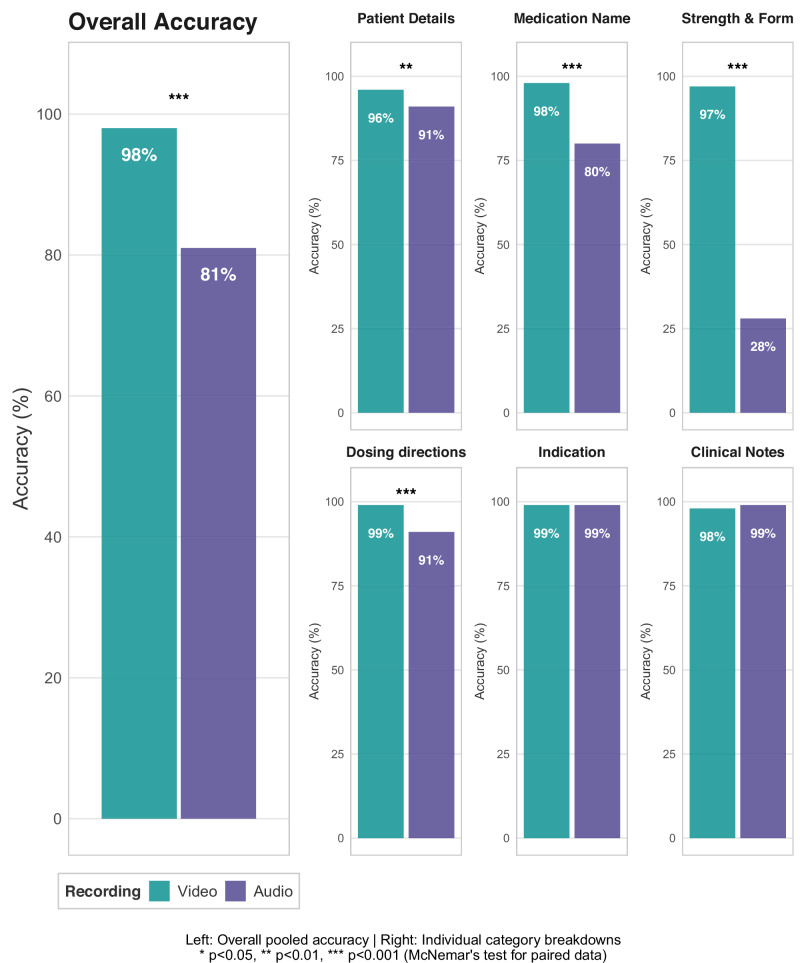
Comparing the accuracy of the multimodal AI scribe in medication history taking in the test set with video versus audio-only input.

**Fig. 3:**
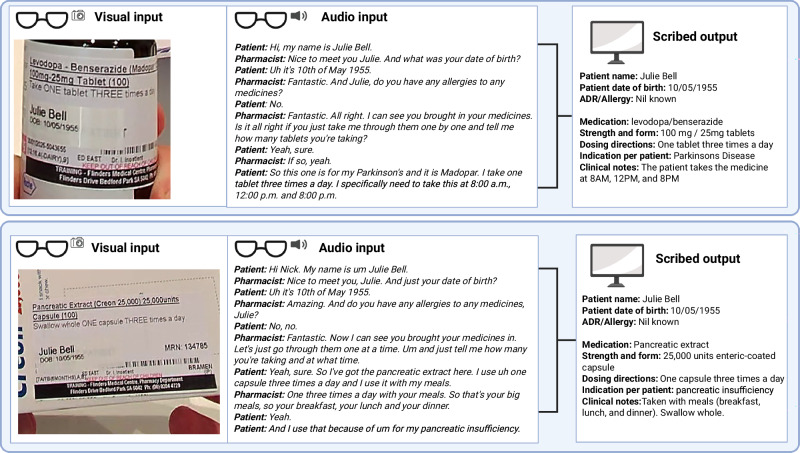
Worked examples of video and audio input being auto scribed by the developed multimodal AI scribe into structured medication history documentation. Created in BioRender. Rowland, A. (2025) https://BioRender.com/0rlac6a.

Overall, the engineered vision-enabled AI scribe correctly scribed 98% (2,114/2,160) of evaluated data points (Table 1, Figs. 1 and 2). This included 96% (288/300) accuracy for patient details, 98% (363/372) for medication name, 97% (362/372) for strength and form, 99% (369/372) for dosing directions, 99% (368/372) for indication, and 98% (364/372) for relevant clinical notes. Accuracy of scribing did not significantly differ according to the pharmacist participating in the interview for dosing directions (range 97% to 100%, *P* = 0.93), indication (range 94% to 100%, P = 0.26), or clinical note (range 93% to 100%, *P* = 0.25) data points. However, significant variation was observed in the accuracy of the AI scribes between pharmacists for patient details (range 80% to 100%, P < 0.001), medication name (range 89% to 100%, P = 0.008), and strength and form (range 86% to 100%, P = 0.009) data (Supplementary Table 1). Figure 3 provides examples of the corresponding model inputs and AI-scribed outputs.

**Table 1:** Comparing the accuracy of the AI scribe in medication history taking in the test set with video versus audio-only input

Category	Video recordings n/N, (%) correct	Audio recordings n/N, (%) correct	P-value^†^
Patient Details	288/300 (96)	272/300 (91)	0.006
Medication Name	363/372 (98)	297/372 (80)	<0.001
Strength and Form	362/372 (97)	105/372 (28)	<0.001
Dose	369/372 (99)	339/372 (91)	<0.001
Indication	368/372 (99)	368/372 (99)	1
Clinical Notes	364/372 (98)	369/372 (99)	0.180
**Pooled total**	**2114/2160 (98)**	**1750/2160 (81)**	**<0.001**

**Definitions:** n/N Number correct out of total possible ^†^ P-value calculated using paired two-tailed McNemar tests.

### Error Classification in the Test Set

Of the 2,160 data points, 46 (2%) were scribed incorrectly by the vision-enabled AI scribe when compared to the human-scribed reference. These included 36 commission (incorrectly scribed content) and 10 omission errors (missing information). Supplementary Tables 2 and 3 display the specific details of the errors made by the AI scribe when processing the video recordings. Supplementary Tables 4 and 5 provide examples of the visual and audio inputs alongside the erroneous outputs.

The 36 commission errors comprised 11 errors in patient details, 8 in medication name, 8 in strength and form, 2 in dosing directions, 2 in indication, and 5 in clinical notes. Among the patient detail commission errors, nine involved incorrect dates of birth, one involved an incorrect name, and one involved an inappropriately recorded allergy, where an allergy to prawns was documented as nil known. For medication names, three errors were due to misidentification of lercanidipine, and five involved incorrect scribing of combination products (e.g., dutasteride/tamsulosin was recorded as solifenacin/tamsulosin). Among the eight strength and form errors, five involved incorrect scribing of combination product strengths, two misrepresented a single-drug product strength (e.g. 145 mg recorded as 160 mg), and one mis-scribed a formulation as “slow-release.” Of the two dosing direction errors, one involved an incorrect time of day for dosing being scribed, and one involved “when required” dosing being recorded as “one or two tablets at night”. The observed indication errors involved a medication prescribed for gastric protection during prednisolone therapy being scribed as for “heartburn”, and a medication for which an indication for pain was scribed, despite not being mentioned in the recording. The five clinical note errors included three cases of incorrect duration of use and two cases where the information in the clinical note conflicted with the dosing directions.

The 10 omission errors (i.e., instances where the AI scribe indicated that a data point was missing when it was actually present) included one involving patient details, one involving medication name, two related to strength and form, one to dosing directions, two to indication, and three to clinical notes. Notably, upon review, all omission errors could be reconciled through manual inspection of the video recording screenshots and the verbatim transcript generated by the AI scribe to support verification in clinical workflows.

### Video Versus Audio Input Comparison

To assess the contribution of visual input to the AI scribe’s accuracy, the 100 test set medication history recordings were reprocessed using audio-only input. The AI scribe correctly scribed 81% (1750 / 2160) of data points when using audio-only input, which was significantly lower than the 98% (2114 / 2160) achieved with video input (P < 0.001; Table 1, Fig. 1, Fig. 2).

Specifically, scribing accuracy was significantly higher with video input compared to audio-only input for patient details (96% vs 91%, P = 0.006), medication name (98% vs 80%, P < 0.001), strength and form (97% vs 28%, P < 0.001), and dosing directions (99% vs 91%, P < 0.001) data. No significant differences were observed between video and audio-only input for indication (99% vs 99%, P = 1.00) or clinical notes (98% vs 99%, P = 0.18) data (Fig. 2, Table 1). The reduction in accuracy with audio-only input was largely due to a substantial increase in omission errors. A total of 358 omission errors occurred with audio-only input, compared to only 10 omission errors with video input (P < 0.001; Supplementary Table 6). Commission errors were also more frequent with the audio-only input (52 errors) compared with the video input (36 errors); however, this difference was not statistically significant (P = 0.063). Supplementary Tables 7 and 8 display the specific details of the errors made by the AI scribe when processing the audio-only input.

**Fig. 4:**
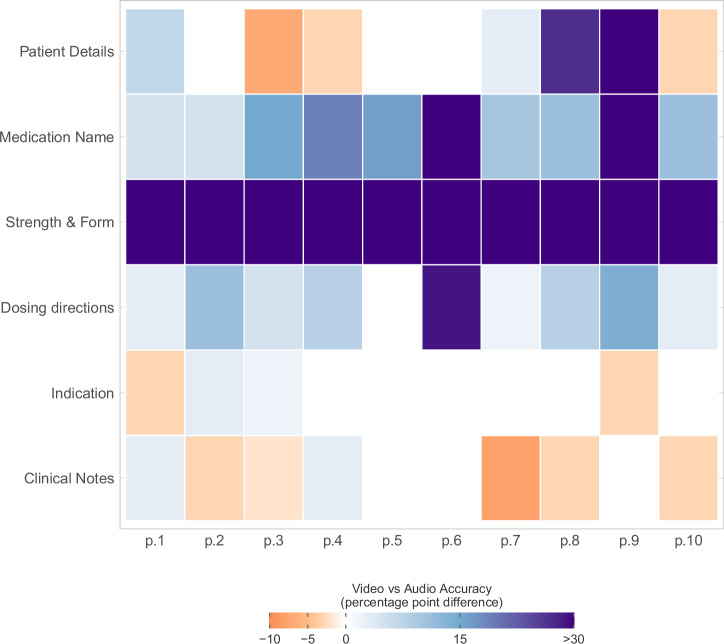
Heatmap of AI scribe accuracy differences between video and audio input by pharmacist and data category. Colours represent percentage point differences (video minus audio), with blue/purple indicating higher accuracy with video input and orange/red indicating higher accuracy for audio input. X-axis represents each of the pharmacists in the study.

Figure 4 presents a heatmap illustrating the absolute change (error rate of the video input minus error rate of the audio-only input) in scribing accuracy for each data field according to each pharmacist when comparing AI scribe performance on audio-only versus video recordings. For all pharmacists, scribing accuracy was consistently higher with video input for medication name (range 5% to 47%), strength and form (35% to 85%), and dosing directions for 9 out of 10 pharmacists (up to 29%) (Fig. 4).

## Discussion

This study developed and evaluated a state-of-the-art vision-enabled AI scribe targeted at enhanced automated documentation of clinical encounters that contain critical visual elements. Medication-history conversations are one example of an information-dense clinical encounter that contains fine-grained details, including the medication name, strength and form, dosing directions, indications, and associated clinical notes. These details are often communicated through both speech and visual cues and may carry immediate and significant clinical consequences when omitted. Across 100 simulated medication histories comprising 2160 data points, the AI scribe achieved an overall accuracy of 98% (2114 of 2160), with accuracy ranging from 96% for patient details to 99% for dosing directions and indication. Of the 46 errors observed, 36 were commission errors, in which the AI scribe documented factually incorrect information. The remaining 10 errors were omissions, where information was marked as missing despite being observable in the video recording. A key finding of the study was that video content substantially improved the accuracy of the AI scribe. Notably, the AI scribe’s performance decreased from 98% with video input to 81% with audio-only input (P < 0.001), driven by a large increase in omissions (358 with audio-only vs 10 with video). Moreover, the accuracy in scribing strength and form decreased from 97% with video recordings to 28% with audio-only recordings. These results highlight the clinical potential of AI scribes paired with video-recording devices to reduce documentation limitations common in audio-only systems, particularly for clinical tasks that require both audio and visual information input.

The accuracy of current proprietary ambient audio-only AI medical scribes (e.g., ScribeMD, DeepScribe, Heidi) is most often reported to range between 82% and 98% for generating clinical consultation notes^17,23,24^. While there is increasing use of these tools and many clinicians report finding them useful, it is important to appreciate that these figures reflect the performance on the spoken information during consultations and therefore overlook the additional work clinicians must perform to incorporate details from written or visual cues to complete the documentation tasks^25^. Illustrating this limitation, a recent study evaluated 14 consultation notes generated by an audio-only AI scribe^26^. Across an average of 400 words per note, more than 20 errors were identified per note, over 80% of which were omissions compared with clinician-scribed, completed references^26^. These findings highlight that with current technologies, clinicians must still spend considerable time reviewing, correcting, and completing AI-generated notes, which is supported by clinician reports in qualitative findings^8,12,27^. Recognising these shortcomings, recent work has sought to integrate visual input into AI scribing workflows to improve completeness and accuracy. For example, an AI scribe trained on surgical video recordings of robotic-assisted radical prostatectomies achieved 87% accuracy in detecting and documenting the surgical steps across 158 tested cases^28^. Similarly, a hybrid vision–language model developed for report generation in laparoscopic cholecystectomies achieved 93% accuracy^29^. Building on these developments, our study presents the first evaluation of a multimodal AI scribe designed for medication history-taking. The system achieved 98% accuracy against human-scribed references across 100 video-recorded encounters, representing a 17% improvement over audio-only processing, with omissions reduced from 358 with audio-only input to 10 with video input. This finding highlights the clinical value of incorporating visual context into documentation tasks that depend on visual communication of information.

While the multimodal AI scribe developed in this study for medication history taking assistance achieved an accuracy of 98% across the 2,160 data points assessed, it is important to appreciate that 46 errors were identified when compared with human-scribed reference. These errors highlight the critical importance of clinician oversight in completing the medication history process. Of the 46 errors, 36 were commission errors, which included inaccuracies in documented patient names and dates of birth, as well as mis-scribing medication names, strengths and forms, dosing directions, indications, and clinical notes. These errors may reflect ambiguity between visually similar products or closely related dose variants (e.g., 145-mg vs 160-mg tablets), while others may have arisen from incomplete capture of visual or spoken details, temporal mismatches between what was said and what was visible, or inherent limitations of the recording device and foundational AI model. Additionally, significant variability in error rates was observed between pharmacists for certain fields, including patient details, medication name, and strength and form, though it remains uncertain whether these differences arose from variation in how information was spoken or visually presented during the simulations or from limitations in the performance of the AI scribe. Left unchecked, such errors could have substantial consequences within downstream clinical workflows impacting patient care. In addition, 10 omission errors were observed, indicating that the scribe technology still has limitations in fully completing documentation, even when the information was observable to human pharmacists from the recordings. Alongside efforts to minimise or eliminate such errors, there is an urgent need for research to establish clinical workflows and protocols that facilitate human-led verification of AI-scribed documents. In the present study, the scribe provided a structured report incorporating selected image frames of medication packaging/labels alongside verbatim transcripts to assist clinicians with efficient verification before document sign-off. Development of robust human-in-the-loop processes remains essential, particularly to mitigate risks of alert fatigue or complacency.

This study utilised a prespecified and prospective test set of 100 simulated medication history recordings featuring ten pharmacists with varied experience, genders, accents, and language backgrounds, across a broad range of clinical scenarios designed to reflect United States disease prevalence data. However, several limitations warrant acknowledgment. With regards to the statistical analysis, although the study was powered prospectively to a small margin of error ( ± 2.5%), it should be noted that multiple comparisons were performed. Further, while the marking of AI outputs was undertaken by two independent evaluators (with discrepancies resolved by a third), the findings may have some clinical subjectivity. The dataset comprised simulated patient encounters enacted by Australian pharmacists speaking the English language with Australian medication packages. Although each pharmacist had experience in conducting medication histories and their “patient” responses were modelled on typical response patterns for the medications used, the artificial nature of the recordings, including that each simulation included the presence of the medicine packages, may not reflect the range of patient behaviours encountered in clinical environments and may reduce the external validity to real-world practice. Additionally, conducting the study in Australia may limit its applicability to international clinical settings and patient populations. Similarly, the medications included in the simulations were weighted toward commonly prescribed agents, owing to the use of disease-prevalence data to guide scenario design. As a result, the study did not extensively evaluate the AI scribe’s performance on newer, less frequently used, off-label, or uncommon medicines. These products along with items in alternative formats, including inhalers, tubes, or compounded preparations or those with older or degraded labels may present greater recognition challenges for the AI scribe. With respect to the accuracy of the scribe processing the audio-only recordings, it is acknowledged that the observed accuracy may increase with further prompt optimisation as conducted with the video recordings. In this study, recordings were collected using Ray-Ban Meta AI Wayfarer glasses under largely optimal lighting and noise conditions, with newly printed labels. In real-world practice, the accuracy of AI scribe may be affected by poor lighting, background noise, overlapping dialogue, and consultations involving a greater number of medicines over extended recording periods^30,31^. Taken together, these factors underscore the need for future research to evaluate the performance of vision-enabled AI scribes across diverse patient populations, clinical environments, and international settings, including those with different languages, while also expanding assessment to a broader range of medication types and dispensing labels.

The multimodal AI model used in this study (Google’s Gemini-Pro-2.5) represented the most advanced foundational generative AI system available for simultaneous audio and video processing at the time of developing the AI scribe^32^. In this study, the workflow involved uploading each medication history recording from the Ray-Ban Meta AI Wayfarer glasses to Google Cloud Storage before scribing, rather than operating in real time. Establishing real-time workflows will therefore be an important focus of future research, which is becoming increasingly feasible with advances in model capability, processing speed, and system integration. Future work should also prioritise understanding the root causes behind the errors observed in vision-enabled AI scribes to guide targeted improvements in reliability and safety. Additionally, accuracy in the AI scribe may also be improved by alternative prompt engineering strategies, emerging models with improved functionality, or by linking to electronic health or pharmacy records, which are central to medication reconciliation in real-world practice and may provide complementary clinical context beyond audio or visual input alone.This study demonstrated that vision-enabled AI scribes can consolidate multiple information sources during medication history taking, reconciling visual cues from medication packaging with spoken exchanges between patients and clinicians. Such capabilities present opportunities towards enhancing documentation completeness, along with transcription efficiency and accuracy. While our evaluation focused on medication-history interviews, the same principles extend to a wide range of visually rich clinical tasks. First, alphanumeric visual information (e.g. patient identification and allergy wristbands, lot or batch numbers, transfusion unit identifiers, infusion rates and modes, and device dose counters) is central to tasks such as verifying patient identity, recording product traceability, and confirming administration parameters. These details cannot be reliably recovered from speech alone, and visual cross-checking by AI may therefore represent a high-value approach to reducing omissions and transcription errors in such tasks. Second, procedural and safety checks, such as verifying drug–dose–patient matches during preparation, confirming vial–syringe correspondence, assessing syringe draw volumes, or pill counts, are also inherently visual and time-critical. Third, patient state and interaction cues - such as visible effort of breathing, pain behaviours, guarded posture, reliance on mobility aids, and salient team–patient exchanges - frequently inform documentation of clinical status. Vision-enabled ambient scribing could capture these cues as structured prompts or candidate text for clinician review.

Moving forward, several measures will be required to ensure the safe and effective adoption of vision-enabled AI scribes into clinical practice. Among the most critical measures are robust safeguards to protect patient privacy and data security^33^, particularly given the sensitive nature of video recordings captured during clinical encounters. Additionally, understanding how the presence of vision-enabled AI scribes may affect the clinician–patient relationship and communication dynamics is essential. For example, patients may feel uncomfortable being recorded or may withhold sensitive information, particularly when visual documentation extends to applications such as recording visible health conditions, appearance or personal behaviours^34,35^. These concerns underscore the importance of engaging patients and stakeholders in implementation planning, particularly regarding informed consent processes. Alternative approaches worth exploring could include still-image capture of corresponding visual clinical elements rather than continuous video recording as a method of preserving privacy. Beyond privacy considerations, successful adoption will require evaluating the costs of system deployment, integration, and maintenance relative to existing workflows, including cost-effectiveness analyses and the potential return on investment. Finally, prospective studies with governance oversight will be needed to support adoption, quantifying the benefits of these tools while monitoring for any emerging risks, including overreliance, biased outputs, technology misuse and potential alert fatigue^36–41^.

In conclusion, this study presents the first development and evaluation of a vision-enabled AI scribe for medication history taking, demonstrating substantial gains over audio-only approaches. Across 100 simulated medication history encounters video recorded using Ray-Ban Meta AI Wayfarer glasses and comprising 2,160 data points, the AI scribe achieved 98% overall accuracy compared with the human-scribed reference, with performance ranging from 96% for patient details to 99% for dosing directions and indication data. Notably, the system performed consistently across the 10 pharmacists evaluated, and the study directly demonstrated that assessment of video input significantly improved accuracy over audio-only evaluations (98% vs 81%, P < 0.001), driven by a marked reduction in omissions (358 with audio-only input vs 10 with video input). These findings underscore the potential value of multimodal AI systems that can interpret visual context for assisting with documentation tasks that are inherently reliant on observation. With further development toward real-time functionality, broader clinical applications, and sustained high accuracy, ambient vision-enabled AI scribes hold substantial potential to transform clinical documentation, reduce administrative burden, enhance clinician–patient interactions, and ultimately improve patient care.

## Methods

### Data Source

Five pairs of research clinical pharmacists (10 in total), featuring a selection of accents, language backgrounds, and genders, used Ray-Ban Meta AI Wayfarer glasses to video record 110 unique simulated medication history interviews. In each simulation, one pharmacist (wearing the glasses) acted as the clinician and the other as the patient. Ten of the simulated medication history recordings were selected to assist in the AI scribe development (training set). The remaining 100 recordings were reserved for AI scribe testing (test set). The test set was not available during AI scribe development and was analysed only once after the AI scribe was developed. Each of the 110 clinical scenarios were unique and preplanned by the authorship team, drawing on their collective clinical experience. The scenarios were designed to reflect common clinical situations based on United States disease-prevalence data, and to include a mix of commonly used prescription and over-the-counter medicines to treat these conditions^42^. Each scenario involved the patient using approximately three to five medicines. Across the simulations, 100 different physical medication containers were used, including boxes or bottles for tablets, capsules, injections, and creams. Each container was labelled using iPharmacy software in accordance with United States Pharmacopeia (USP) standards^43^. Interview scenarios also included references to non-present products, such as over-the-counter or as-needed medicines and supplements. All recordings were conducted in quiet meeting rooms with standard fluorescent lighting, with the two pharmacists seated across from each other. A member of the research team (B.D.M.) was present to provide technical support as needed. All 110 recordings were stored in Google Cloud Storage prior to analysis.

For each recorded interview, a human-scribed reference was created by B.D.M., a clinical pharmacist, to serve as the ground truth for comparison. Each history was structured according to recommended documentation standards and included patient-level details (name, date of birth, and medication allergies) and medication-level details (medication name, strength and form, dosing directions, indication, and relevant clinical notes)^44,45^. All human-scribed medication histories were independently reviewed and verified for accuracy by a second clinical pharmacist (N.S.), with a third pharmacist (A.M.H.) to resolve discrepancies.

### AI Scribe Development

The vision-enabled AI scribe was developed using Google’s Gemini-Pro-2.5-exp-05-06, a multimodal foundation model selected for its strong performance and ability to process both audio and video input^32^. The scribe was engineered using validated prompt engineering strategies^46,47^, including guidance on testing, task decomposition, example inclusion, structured output formatting, and output verification. Prompts were iteratively refined using the 10 training video recordings to maximise accuracy (Fig. 1).

Briefly, the engineered prompts assigned the model the task of scribing a medication history interview, broken into steps for documenting patient details (name, date of birth, and medication allergies) and medication-specific data (medication name, strength and form, dosing directions, indication, and relevant clinical notes). The model was instructed to return this information in a structured JavaScript Object Notation (JSON) format. A second prompt was used to verify the output by providing the original video alongside the draft output, instructing the model to identify and correct any errors or omissions. Additional strategies included providing input–output examples (i.e. medication history recordings and their correct outputs), instructions to generate screenshots of the medication box/label and a verbatim transcript to assist verification within clinical workflows.

The scribe was iteratively trained until no further accuracy improvements were observed in the training data. Final prompt configurations are provided in the Supplementary information. Hyperparameters were set to a temperature of 0.0, a top_p of 0.95, and a maximum output length of 16,000 tokens to ensure deterministic outputs. Development and testing were conducted between January and May 2025 using Python (v3.12) within Visual Studio Code (v1.99.1). The AI Scribe system, including all optimised prompts used during training with the video recordings, was frozen before the unblinding and evaluation of the test set.

### AI Scribe Testing

The performance of the developed AI medication history scribe was evaluated using the 100 test set medication history interview recordings. For each interview, the output generated by the AI scribe was compared against the human-scribed reference (ground truth). Accuracy was assessed across patient-level fields (name, date of birth, and medication allergies- recorded once per interview) and medication-level fields (medication name, strength and form, dosing directions, indication, and clinical notes- recorded for each medicine mentioned in the interview). Relevant clinical notes included any visual or spoken information contained within the recording that would typically be documented within a medication history as it relates to the use of a medication (e.g., administration details, recent changes, duration of use). Each data field was classified as correct or incorrect. Incorrect entries were further categorised into omission or commission errors as defined in the literature for documenting medication errors^48,49^. Omission errors occurred when a required field was absent/unknown in the AI output despite being present in the human-scribed reference, whereas commission errors occurred when a value was present but was discordant with the reference. These terms describe disagreement with the pharmacist scribed reference based on the video recordings, and do not attribute the root cause of the error. Entries in the clinical notes field that conveyed additional information beyond the human-scribed reference were considered correct, provided the information was identifiable in the video or audio recording and did not conflict with any of the other fields. Two clinical pharmacists (B.D.M. and N.S.) independently completed the reviews, and discrepancies were resolved by a third pharmacist (A.M.H.).

All analyses and visualisations were conducted in RStudio (version 5.1), with statistical significance set at *P* < 0.05. The accuracy of the AI scribe was examined using descriptive statistics and presented with bar plots. It was estimated that a test set of 100 video recordings, yielding over 300 data points for a field type. (i.e. patient-level, medication name, strength and form, dosing directions, indication, and clinical note data fields), had a predicted margin of error of ±2.5% on a predicted accuracy of 95% for the field (i.e., an observed accuracy of 95% for the field would result in a 95% CI of 92.5% to 97.5%).

For the secondary aim, the same optimized prompts used to process the video recordings were applied to the audio-only versions of each recording. Descriptive statistics and paired McNemar’s tests were used to assess differences in scribe performance between video and audio-only input. Assuming a discordant rate of ≤10%, consistent with the expectation that the two modalities would usually agree, >2000 paired data points provides >90% power (two-sided McNemar test, α = 0.05) to detect an absolute accuracy difference of ≥5 percentage points. A heat map was used to visualise differences in scribe accuracy between pharmacists when processing the video versus audio-only.

This study was approved by the Flinders University Human Research Ethics Committee (Project ID: 7800), in accordance with the National Statement on Ethical Conduct in Human Research by the National Health and Medical Research Council, Australia. Consent to participate: The study did not involve human participants; therefore, informed consent was not applicable.

## Supplementary information

Supplementary Information

## Data Availability

All data, including the patient and medication details used in the simulated cases and the corresponding AI outputs, are available at https://zenodo.org/records/17032178.

## Code availability

The Python code used to create the vision enabled AI scribe is available at [Vision-Enabled-AI-Scribe] ([https://github.com/MenzBD/Vision-Enabled-AI-Scribe/tree/main]). The R scripts used to generate the manuscript and supplementary tables and figures are available at [Vision-Enabled-AI-Scribe] ([https://github.com/MenzBD/Vision-Enabled-AI-Scribe/tree/main]). The data file is restricted to public access, and is available to the reviewers or editor(s) while under peer review.
