# Supplementary material for: Vision-Enabled AI scribes reduce omissions in clinical conversations: evidence from simulated medication histories
Source: NPJ Digit Med. 2026 Feb 26;9:287. doi: 10.1038/s41746-026-02494-9 (PMC13057267; doi:10.1038/s41746-026-02494-9)
Supplement: Supplementary file 1 — Supplementary Information [file 41746_2026_2494_MOESM1_ESM.pdf]

Supplementary information

Supplementary file for BD. Menz, NL. Scarfo, ND. Modi, E. Cornelisse, LX. Li, JQE. Tan, J. Gandhi, D. Maher, D. Kousa, K. Daniel, V. Menon, S. Bacchi, RA. McKinnon, MD. Wiese, A. Rowland, MJ. Sorich, AM. Hopkins, '*Vision Enabled AI Scribes Reduce Omissions in Clinical Conversations: Evidence from Simulated Medication Histories*'

This supplementary has been provided by the authors to give readers additional information about the work.

Contents

**Prompt 1:**<br>Initial extraction	**Page 3**
**Prompt 2:**<br>Extraction verification	**Page 5**
**Prompt 3:**<br>Video to image timestamp	**Page 7**
**Prompt 4:**<br>Audio transcription	**Page 8**
**Supplementary table 1.**<br>Accuracy of the AI scribe processing the video recordings between pharmacists	**Page 9**
**Supplementary table 2.**<br>Commission errors by the AI scribe when processing the video recordings.	**Page 10**
**Supplementary table 3.**<br>Omission errors by the AI scribe when processing the video recordings.	**Page 12**
**Supplementary table 4.**<br>Example commission errors identified in the video recordings.	**Page 13**
**Supplementary table 5.**<br>Example omission errors identified in the video recordings	**Page 14**
**Supplementary table 6.**<br>Frequency of commission and omission errors by AI scribe when processing the video and audio recordings	**Page 15**
**Supplementary table 7.**<br>Commission errors by the AI scribe when processing the audio recordings..	**Page 16**

**Supplementary table 8.**<br>Omission errors by the AI scribe when processing the audio recordings.	**Page 18**

Prompt 1: Initial extraction

You are tasked with transcribing and extracting structured information from a medication history interview conducted by a clinical pharmacist. The source material may include audio-only or video recordings of a conversation between a pharmacist and a patient. Your objective is to identify and document all relevant medication-related information conveyed during the interaction. This includes both spoken dialogue and any visible written materials such as medication labels, packaging, or charts captured in the video. The information must be extracted and formatted precisely according to the step-by-step instructions outlined below.

Step 1: Document the following patient and pharmacist details:

- The pharmacist's name.
- The patient's name (first and last name), date of birth (dd/mm/yyyy), and any medicine allergies (document the generic medicine name and the reaction. If a patient reports no allergies, specify this as Nil known).

Step 2: Document a complete and accurate list of the patient's regular medications, including:

- The generic name of the medicine (example: metformin, ramipril, epoetin lambda). If the medicine is in a combination, ensure that you return both medicines separated by a forward slash.
- The medicine strength and form (example: 500 mg tablets, 200 mg/5 mL syrup, 8000 unit injection). If the medicine is in a combination ensure you return both strengths separated by a forward slash. If the medicine is a slow-release or controlled-release preparation, please ensure you also document this.
- The directions for each medicine, expressed as the number of units and the frequency of the medicine taken. Example: One tablet every morning, one tablet twice a day, one tablet at night. Do not include any other details.
- What the patient reports taking each of the medicines for (the indication). Document this using medical nomenclature. Example: hypertension, diabetes, infection.

Step 3: Document any clinical information related to the medication, including:

- If the patient crushes or modifies the tablet or capsule. Example: the patient crushes the tablet and mixes it with yogurt.
- If specific dosing times are mentioned. Example: the patient takes the medicine at 8 AM and 8 PM, as instructed by their cardiologist.
- If the patient requires assistance to administer the medicine. Example: the patient's wife administers the injection.
- If the patient has difficulty handling medication due to dexterity issues. Example: the patient uses a pill cutter to split the tablet before taking it.
- If there are recent medicine changes or discrepancies clarified by the pharmacist. Example: the patient stated that the medicine was started this week.

- If there is any associated monitoring with the medicine. Example: the patient has their calcium monitored each week by their family physician.
- If no additional details are relevant, return (missing).

Step 4: Document the source of the information:

- audio only when information was only spoken by the patient or pharmacist.
- visual only when information was only found on the medication box or dispense label.
- audio+visual when information is identical in both audio and visual sources.
- If there is ambiguity between the audio and visual cues, include both interpretations in your response using the keys audio and visual. Example: audio: <value> and visual: <value>.

Step 5: Return the extracted information in the following structured JSON format:

```
{
"pharmacist": "<pharmacist_name>",
"name": "<patient_name>",
"dob": "<date_of_birth>",
"allergies": "<allergies>",
"medications": [
{
"medicine": "<generic_name>",
"strength_and_form": "<strength_and_form>",
"dose": "<dose_summary>",
"indication": "<indication_per_patient>",
"clinical_notes": "<clinical_notes_for_this_medication>",
"source": "<audio only|visual only|audio+visual>"
},
...
]
}
```

Final Directive: Please ensure that your final output is strictly the JSON object described above, with no additional commentary.

Final Directive: Please ensure that your final output strictly matches the JSON object described above, capturing audio and visual verbatim segments for each field, even if marked as missing. Do not include any additional commentary or explanations.

Prompt 2: Extraction verification

You are a medication history verification agent. Your task is to review a structured medication history that was previously extracted from a pharmacist–patient interview recorded as audio or video. Your responsibility is to ensure that each item in the medication history is fully and accurately supported by the recording.

This is Part 2 of a two-step process. In Part 1, structured information—such as medication names, strengths, doses, and indications—was extracted directly from the recording. In this Part 2 task, you must critically verify the accuracy of the medication history against the original recording, correct any inaccuracies, and ensure the final medication history is returned in the JSON format specified in Part 1.

Below is the step-by-step process for verifying the accuracy of the extracted medication history.

Step 1: Verify the accuracy of the extracted medication information

Review each piece of information in the extracted medication history and confirm it matches the recording. For each medication, you must check:

- Medicine name: Ensure the medication name matches exactly what was mentioned in the recording
- Strength and form: Verify the medication strength and form (tablets, syrup, etc.) are correct
- Dose instructions: Check that the dosing frequency and timing match the recording
- Indication: Confirm the reason for taking the medication is accurately recorded
- Clinical notes: Verify any additional details about how the patient takes or handles the medication

If any inaccuracies are identified, you must correct them. Here are some examples:

- Wrong medicine name: If the medication history shows 'simvastatin' but the recording clearly states 'atorvastatin', correct the entry to 'atorvastatin'
- Wrong dosing: If the medication history shows 'one tablet in the morning' but the recording states 'one tablet at night', change the entry to match the recording
- Missing medicines: If the patient mentioned taking paracetamol when needed, but this doesn't appear in the medication history, add it as a new medication entry

Step 2: Check for missing or duplicate medications

- Missing medications: Ensure all medications mentioned by the patient are included in the final medication history
- Duplicate entries: If the same medication appears more than once in the medication history, combine the entries into a single, complete entry

Step 3: Return the corrected medication history

Use the exact same JSON structure and field names as specified in Part 1. If any information cannot be verified from the recording, mark that field as "(missing)". Return only the corrected JSON object with no additional text or explanations.

Final directive

Always strictly base your review on the recording provided. Never invent, infer, or guess missing information. If you cannot verify a field with certainty, mark it as '(missing)'. Apply this rule consistently across all fields including medicine names, strengths, forms, dosages, indications, and clinical notes.

For reference, these are the original instructions provided in part 1.

[Insert part one here]

Here is the extracted JSON of the medication history extracted in part 1.

[Insert extracted JSON object of medication here]

Here is the recording I want you to review:

[Insert medication history recording here]

Prompt 3: Video to image timestamp

You are tasked with reviewing a recorded medication history interview between a clinical pharmacist and a patient. Your goal is to identify the exact timestamps when medication dispensing labels—visible on boxes, bottles, or other packaging—are most clearly shown in the recording. These timestamps will be used for documentation and verification purposes.

Step 1: Determine the two clearest timestamps for each medication
For each medication identified in the recording, locate two timestamps where:

- The dispensing label is fully visible and unobstructed
- The medication name, including both brand and generic names (if shown), is clearly visible
- Label details such as strength, directions, and patient name are legible
- The image quality is suitable for accurate transcription
- The two timestamps should capture the clearest possible views

Step 2: Report your findings using the following format

- Format the time expressed as MM:SS (example: 12:34)
- Round each timestamp to the nearest second where label clarity is highest
- Report your findings as Medication Name: [Name as shown on label or state 'Unknown if illegible', Clearest Timestamps: [First timestamp] and [Second timestamp]

Final directive
If no medication containers with clearly readable labels are visible in the recording, respond with exactly:
No clear medication labels found.
Do not include any guesses or assumptions based on unclear or partial label views.

Prompt 4: Audio transcription

You are tasked with transcribing a recorded medication history interview between a clinical pharmacist and a patient. Your goal is to provide a complete and accurate verbatim transcription of all spoken dialogue for clinical documentation purposes.

Step 1: Transcribe all spoken words in the exact sequence they occur, this includes:

- Every question asked by the pharmacist
- Every response from the patient
- All clarifications, corrections, or follow-up statements by either party
- Medication names, dosages, and directions exactly as spoken, even if mispronounced

Step 2: Ensure accuracy of the transcribed record

- Do not correct mispronunciations or incomplete terms
- Include all numerical details such as dose, timing, and dates
- Accurately transcribe all medical conditions or terminology
- If a word is partially audible, include your best interpretation followed by the word 'unclear' in square brackets

Step 3: Use speaker tags for clarity

- Begin each new line with either 'pharmacist' or 'patient' followed by a colon, depending on who is speaking
- Start a new line for every change in speaker
- Do not insert any of your own commentary or interpretations

Final directive
Return only the verbatim transcript of the dialogue with clear speaker tags.
Do not summarize, paraphrase, or omit any part of the conversation.
Precision and completeness are essential for clinical accuracy.

Supplementary table 1. Accuracy of the AI scribe between pharmacists

Pharmacist	Patient Details	Medication Name	Strength & Form	Dosing directions	Indication	Clinical Notes
p.1	30/30, (100.0%)	39/39, (100.0%)	39/39, (100.0%)	39/39, (100.0%)	38/39, (97.4%)	39/39, (100.0%)
p.2	30/30, (100.0%)	37/37, (100.0%)	37/37, (100.0%)	37/37, (100.0%)	37/37, (100.0%)	36/37, (97.3%)
p.3	24/30, (80.0%)	40/40, (100.0%)	40/40, (100.0%)	39/40, (97.5%)	40/40, (100.0%)	39/40, (97.5%)
p.4	28/30, (93.3%)	33/37, (89.2%)	35/37, (94.6%)	37/37, (100.0%)	36/37, (97.3%)	37/37, (100.0%)
p.5	29/30, (96.7%)	37/37, (100.0%)	36/37, (97.3%)	37/37, (100.0%)	37/37, (100.0%)	37/37, (100.0%)
p.6	30/30, (100.0%)	34/34, (100.0%)	34/34, (100.0%)	34/34, (100.0%)	34/34, (100.0%)	34/34, (100.0%)
p.7	30/30, (100.0%)	40/40, (100.0%)	39/40, (97.5%)	39/40, (97.5%)	40/40, (100.0%)	37/40, (92.5%)
p.8	28/30, (93.3%)	35/36, (97.2%)	35/36, (97.2%)	36/36, (100.0%)	36/36, (100.0%)	34/36, (94.4%)
p.9	30/30, (100.0%)	35/36, (97.2%)	36/36, (100.0%)	36/36, (100.0%)	34/36, (94.4%)	36/36, (100.0%)
p.10	29/30, (96.7%)	33/36, (91.7%)	31/36, (86.1%)	35/36, (97.2%)	36/36, (100.0%)	35/36, (97.2%)
P-value	P<0.001	P=0.008	P=0.009	P=0.933	P=0.256	P=0.249
P-value calculated using chi-square tests.<br>Accuracy expressed as n/N: number correct / total number						

Supplementary table 2. Commission errors by the AI scribe when processing the video recordings.

Category	Pharmacist scribed	AI scribed	Error details
Patient Details	10/09/1955	10/09/1992	Incorrect patient details
Patient Details	10/09/1955	10/09/1965	Incorrect patient details
Patient Details	10/09/1955	10/09/1965	Incorrect patient details
Patient Details	10/09/1955	10/09/1985	Incorrect patient details
Patient Details	Prawns	Nil known	Incorrect patient details
Patient Details	15/01/1970	15/01/1972	Incorrect patient details
Patient Details	15/01/1970	13/01/1970	Incorrect patient details
Patient Details	20/09/1975	28/09/1975	Incorrect patient details
Patient Details	Missing	18/10/1985	Incorrect patient details
Patient Details	Lesley smart	Leslie Smart	Incorrect patient details
Patient Details	15/05/1970	15/05/1972	Incorrect patient details
Medication Name	Dutasteride / tamsulosin	solifenacin/tamsulosin	Incorrect medication
Medication Name	Clotrimazole / hydrocortisone	hydrocortisone/clotrimazole/neomycin	Incorrect medication
Medication Name	lercanidipine	lansoprazole	Incorrect medication
Medication Name	Lercanidipine	lisinopril	Incorrect medication
Medication Name	Ferrous fumerate / folic acid	ferrous fumarate/folic acid/ascorbic acid	Incorrect medication
Medication Name	Ferrous fumerate / folic acid	audio: iron tablets, visual: ferrous sulfate/ascorbic acid	Incorrect medication
Medication Name	Lercanidipine	audio: Zircol 20, visual: lisinopril	Incorrect medication
Medication Name	Purple puffer, potentially Seretide	fluticasone/salmeterol	Incorrect medication
Strength & Form	500 microg / 400 microg slow release capsules	6 mg/400 microgram slow release capsule	Incorrect strength or form
Strength & Form	1% / 1% cream	1%/1%/0.5% cream	Incorrect strength or form
Strength & Form	60 mg slow release tablets	60 mg tablet	Incorrect strength or form
Strength & Form	310 mg / 350 microg	310 mg/350 mcg/500 mg tablets	Incorrect strength or form
Strength & Form	145 mg tablet	160 mg tablets	Incorrect strength or form
Strength & Form	310 mg / 350 microg tablets	audio: (missing), visual: 325 mg/500 mg controlled release tablet	Incorrect strength or form
Strength & Form	25 mg tablets	20 mg tablet	Incorrect strength or form
Strength & Form	probably 250 microg	250 mcg/25 mcg inhaler	Incorrect strength or form
Dosage	One tablet when I want to	One or two tablets at night	Incorrect dose
Dosage	One tablet at night	One tablet daily	Incorrect dose
Indication	Taken to protect the stomach while on prednisolone	heartburn	Incorrect indication
Indication	Missing	Pain	Incorrect indication
Clinical Notes	Patient guesses their symptoms are under control	The patient reports taking this medicine 'when I want to', which differs from the labelled dose of 'One or two tablets at night'. The patient also expressed doubt about their diagnosis of schizophrenia.	Incorrect clinical note
Clinical Notes	Has been taking for roughly one week	The patient stated that the medicine was started this week.	Incorrect clinical note
Clinical Notes	Psychiatrist started me on last week	The patient stated that the medicine was started this week.	Incorrect clinical note
Clinical Notes	Started a week prior	The patient stated that the medicine was started this week. Course completed. Patient swallows tablets whole.	Incorrect clinical note

Clinical Notes	Missing	The patient takes one tablet at night when required. The pharmacist advised the patient can adjust the dose as needed.	Incorrect clinical note

Supplementary table 3. Omission errors by the AI scribe when processing the video recordings.

Category	Pharmacist scribed	AI scribed	Error details
Patient Details	Nil known	(missing)	Patient details missing
Medication Name	Athletes foot cream, brand / drug unknown	(missing)	Medication missing
Strength & Form	1 g (missing)	(missing)	Strength or form missing
Strength & Form	50 mg / 8 mg tablets	Tablets	Strength or form missing
Dosage	When I need it, with my Panadol	(missing)	Dose missing
Indication	Heart failure	Missing	Indication missing
Indication	For the heart	Missing	Indication missing
Clinical Notes	Dissolved on the tongue	Missing	Clinical note missing
Clinical Notes	Before bed	Missing	Clinical note missing
Clinical Notes	Uses for a while then rash goes away	Missing	Clinical note missing

Supplementary table 4. Example commission errors identified in the video recordings.

Error detail	Video input	Audio input	Model output
Wrong medication name	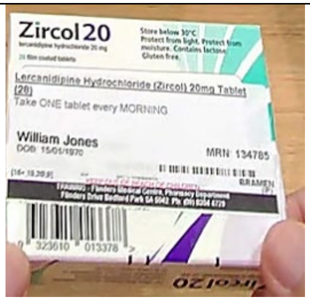	<u>*Pharmacist:*</u><br>*And how about this one here? The Zircol?*<br><u>*Patient:*</u><br>*So, um, one in the morning.*<br><u>*Pharmacist:*</u><br>*Okay. And then what's that one for?*<br><u>*Patient:*</u><br>*So for blood pressure.*	<u>**Pharmacist scribed output**</u><br>Medication: lercanidipine<br><br><u>**Scribe output**</u><br>Medication: lisinopril
Wrong strength and form	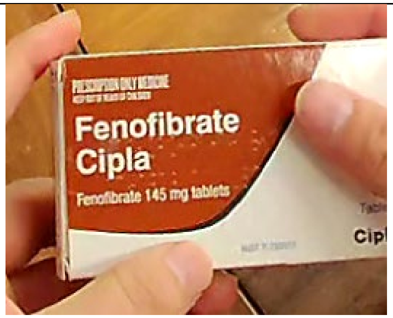	<u>*Patient:*</u><br>*And then I'm on Fenofibrate.*<br><u>*pharmacist:*</u><br>*Okay.*<br><u>*pharmacist:*</u><br>*And are you taking one tablet in the morning with this one?*<br><u>*patient:*</u><br>*Yes.*	<u>**Pharmacist scribed output**</u><br>Strength and form: 145 mg tablets<br><br><u>**Scribe output**</u><br>Strength and form: 160 mg tablets
Wrong medication name	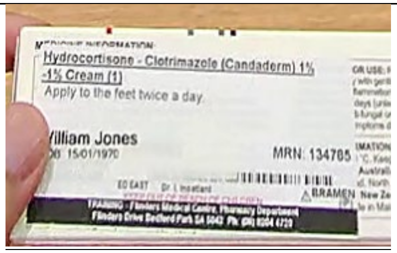	<u>*Pharmacist:*</u><br>*Yeah. And how about this last one here, this cream?*<br><u>*Patient:*</u><br>*I got a um rash on my foot. So I just apply it twice a day.*<br><u>*Pharmacist:*</u><br>*Right. How long have you been uh using it for?*<br><u>*Patient:*</u><br>*Uh just for the last few days.*	<u>**Pharmacist scribed output**</u><br>Clotrimazole / hydrocortisone<br><br><u>**Scribe output**</u><br>Hydrocortisone/clotrimazole/neomycin

Supplementary table 5. Example omission errors identified in the video recordings

Error detail	Video input	Audio input	Model output
Missing clinical notes	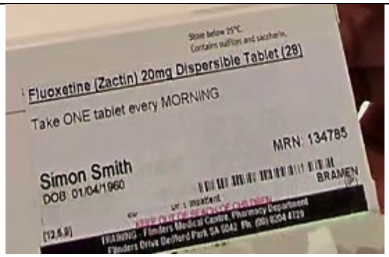	<u>*Pharmacist:*</u><br>*Okay, great. Um now, now I wanted to see you because I wanted to go through your medications. Um do you want to just tell me what you take and exactly what the dosage and instructions are?*<br><u>*Patient:*</u><br>*Abs- absolutely. Um I might start off with my Zactin. So I take one of these um tablets which disperse on my tongue. I take one of these in the morning for my depression.*	<u>**Pharmacist scribed output**</u><br>Dissolved on the tongue<br><br><u>**Scribe output**</u><br>Missing
Missing indication	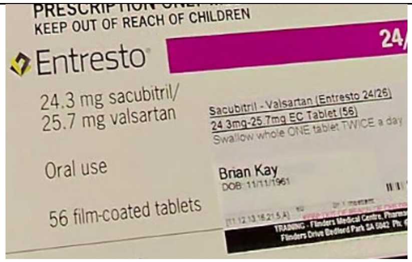	<u>*Patient:*</u><br>*Uh, so the first one I'm taking is called Entresto.*<br><u>*Pharmacist:*</u><br>*Mhm.*<br><u>*Patient:*</u><br>*Uh and I take one of these twice a day.*<br><u>*Pharmacist:*</u><br>*Okay. And do you know why you're taking this one?*<br><u>*Patient:*</u><br>*Uh, the doctor told me I've got heart failure.*	<u>**Pharmacist scribed output**</u><br>Heart failure<br><br><u>**Scribe output**</u><br>Missing
Missing clinical notes	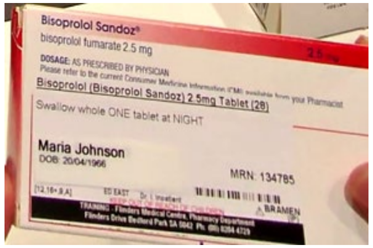	<u>*Pharmacist:*</u><br>*One at night time. All right. Um next we've got Bisoprolol.*<br><u>*Patient*</u>*: Mhm.*<br><u>*Pharmacist:*</u><br>*Uh do you know what you take this for?*<br><u>*Patient:*</u><br>*Um for my heart problems.*<br><u>*pharmacist:*</u><br>*Heart problems. And how many of these do you take?*<br><u>*Patient:*</u><br>*Take one before bedtime.*<br><u>*Pharmacist:*</u><br>*One at bedtime. Okay, great.*	<u>**Pharmacist scribed output**</u><br>Before bed<br><br><u>**Scribe output**</u><br>Missing

Supplementary table 6. Frequency of commission and omission errors by AI scribe when processing the video and audio recordings

Category	Count	Omissions			Commissions		
		**Audio-video**	**Audio**	**P†**	**Audio-video**	**Audio**	**P†**
Patient Details	300	1	4	0.37	11	24	0.013
Medication Name	372	1	58	<0.001	8	17	0.067
Strength and Form	372	2	267	<0.001	8	0	0.004
Dose	372	1	24	<0.001	2	9	0.033
Indication	372	2	3	1	2	1	0.32
Clinical Notes	372	3	2	1	5	1	0.10
**Total Counts**	**2160**	**10**	**358**	**<0.001**	**36**	**52**	**0.063**

Definitions: Omission - failure to scribe an item that was present in the clinical interview. Commission - scribing an item that was not present in the clinical interview or scribing an item incorrectly. † P-value calculated using McNemar's test for paired error type comparisons.

Supplementary table 7. Commission errors by the AI scribe when processing the audio recordings.

Category clean	Pharmacist scribed	Scribed	Outcome detail
Clinical Notes	Has been taking for roughly one week	The patient stated that the medicine was started this week.	Incorrect clinical note
Dosing directions	One capsule three times a day	One tablet three times a day	Incorrect dose
Dosing directions	One tablet at night	One tablet when needed for sleep	Incorrect dose
Dosing directions	One capsule every morning	One tablet every morning	Incorrect dose
Dosing directions	One capsule in the morning	One tablet every morning	Incorrect dose
Dosing directions	One application to the feet twice a day	Apply twice a day	Incorrect dose
Dosing directions	One capsule in the morning	Once every morning	Incorrect dose
Dosing directions	One capsule in the morning	One tablet every morning	Incorrect dose
Dosing directions	One tablet twice a day	One capsule twice a day	Incorrect dose
Dosing directions	One capsules twice a day	One tablet twice a day	Incorrect dose
Medication Name	Indapamide	Insec	incorrect medication
Medication Name	Epoetin lamda	epoetin alfa	incorrect medication
Medication Name	Isosorbide mononitrate	Duret	incorrect medication
Medication Name	Lercanidipine	metolazone	incorrect medication
Medication Name	Isosorbide mononitrate	Du-ride	incorrect medication
Medication Name	Verapamil	Anpec	incorrect medication
Medication Name	Irbesartan	erbesartan	incorrect medication
Medication Name	Sildenafil	tadalafil	incorrect medication
Medication Name	Ferrous fumerate / folic acid	Ferrous preparation	incorrect medication
Medication Name	Bisoprolol	sotalol	incorrect medication
Medication Name	Atorvastatin	losartan	incorrect medication
Medication Name	Panamax	paracetamol/codeine	incorrect medication
Medication Name	Ferrous fumerate / folic acid	iron	incorrect medication
Medication Name	Epoetin lambda	epoetin alfa	incorrect medication
Medication Name	Sildenafil	metoprolol	incorrect medication
Medication Name	Purple puffer, potentially seretide	fluticasone/salmeterol	Incorrect medication
Medication Name	Verapamil	Ampec	incorrect medication
Indication	Missing	diabetes	Incorrect indication
Patient Details	Julie Bell	Julia Bell	Incorrect patient details
Patient Details	10/09/1955	10/09/1985	Incorrect patient details
Patient Details	10/09/1955	10/09/1965	Incorrect patient details
Patient Details	15/01/1970	13/01/1970	Incorrect patient details
Patient Details	20/09/1975	20/09/1935	Incorrect patient details
Patient Details	Lesley smart	Leslie Smart	Incorrect patient details
Patient Details	Lesley smart	Leslie Smart	Incorrect patient details
Patient Details	Lesley smart	Leslie Smart	Incorrect patient details
Patient Details	Lesley smart	Leslie Smart	Incorrect patient details
Patient Details	Lesley smart	Leslie Smart	Incorrect patient details
Patient Details	Lesley Smart	Leslie Smart	Incorrect patient details
Patient Details	Lesley Smart	Leslie Smart	Incorrect patient details
Patient Details	Lesley smart	Leslie Smart	Incorrect patient details

Patient Details	Lesley smart	Leslie Smart	Incorrect patient details
Patient Details	Maria Johnson	Marie Johnson	Incorrect patient details
Patient Details	Brian Kay	Brian K	Incorrect patient details
Patient Details	Brian Kay	Brian K	Incorrect patient details
Patient Details	Brian Kay	Brian K	Incorrect patient details
Patient Details	Brian Kay	Brian K	Incorrect patient details
Patient Details	Brian Kay	Brian K	Incorrect patient details
Patient Details	Brian Kay	Brian K	Incorrect patient details
Patient Details	Brian Kay	Brian K	Incorrect patient details
Patient Details	Brian Kay	Brian K	Incorrect patient details
Patient Details	Brian Kay	Brian K	Incorrect patient details

Supplementary table 8. Omission errors by the AI scribe when processing the audio recordings.

Category clean	Pharmacist scribed	Scribed	Outcome detail
Clinical Notes	Taken instead of statins	Missing	Clinical note missing
Clinical Notes	Recently started	Missing	Clinical note missing
Dosing directions	One tablet in the morning	(missing)	Dose missing
Dosing directions	One tablet twice a day	One (form not specified) twice a day	Dose missing
Dosing directions	One tablet at night	(Quantity not specified) at night	Dose missing
Dosing directions	One tablet in the morning	One (form not specified) every morning	Dose missing
Dosing directions	Apply to the elbow at night	Apply sparingly to elbow (frequency missing)	Dose missing
Dosing directions	One tablet in the morning	(missing) every morning	Dose missing
Dosing directions	One tablet in the morning	(missing) every morning	Dose missing
Dosing directions	One tablet twice a day	(missing) tablets twice a day	Dose missing
Dosing directions	One tablet twice a day	(missing) units twice a day	Dose missing
Dosing directions	One tablet in the morning	(missing unit) once in the morning	Dose missing
Dosing directions	One tablet twice a day	(missing) twice a day	Dose missing
Dosing directions	One capsule twice a day	(missing) twice a day	Dose missing
Dosing directions	One tablet twice a day	(quantity missing) twice a day	Dose missing
Dosing directions	One tablet twice a day	(missing) twice a day	Dose missing
Dosing directions	One tablet at night	(missing) at night	Dose missing
Dosing directions	One injection twice a day	(missing)	Dose missing
Dosing directions	One application to the elbow at night	Apply at night	Dose missing
Dosing directions	(missing) three times a day	(units missing) three times a day	Dose missing
Dosing directions	One tablet at night	(missing)	Dose missing
Dosing directions	One tablet three times a day	One (unit) three times a day	Dose missing
Dosing directions	One tablet in the morning	One (missing form) every morning	Dose missing
Dosing directions	One tablet twice a day	(missing)	Dose missing
Dosing directions	10 mL three times a day	One dose three times a day	Dose missing
Dosing directions	One capsule with meals, three times a day	Number of units not specified, with meals	Dose missing
Indication	Stroke	Missing	Indication missing
Indication	Pain	Missing	Indication missing
Indication	Schizoprenia	Missing	Indication missing
Medication Name	Esomeprazole	(missing)	Medication missing
Medication Name	ferrous fumerate / folic acid	iron	Medication missing
Medication Name	Fluvoxamine	Unspecified antidepressant	Medication missing
Medication Name	Amiodarone	(missing)	Medication missing
Medication Name	Rosuvastatin	(missing)	Medication missing
Medication Name	Enoxaparin	(missing)	Medication missing
Medication Name	Amoxicillin	Unspecified antibiotic	Medication missing
Medication Name	Simvastatin	Unspecified cholesterol medicine	Medication missing
Medication Name	Atorvastatin	(Not specified)	Medication missing
Medication Name	Bisoprolol	(Not specified)	Medication missing
Medication Name	Perindopril	(Not specified)	Medication missing
Medication Name	Bisoprolol	(missing)	Medication missing

Medication Name	Venlafaxine	Unknown	Medication missing
Medication Name	Dutasteride / tamsulosin	(missing)	Medication missing
Medication Name	Clotrimazole / hydrocortisone	(missing)	Medication missing
Medication Name	lercanidipine	(missing)	Medication missing
Medication Name	Epoetin lambda	(missing)	Medication missing
Medication Name	Amoxicillin / clavulanic acid	Unknown antibiotic	Medication missing
Medication Name	Enoxaparin	(missing)	Medication missing
Medication Name	Amoxicillin / clavulanic acid	Antibiotic (generic name not specified)	Medication missing
Medication Name	Carvedilol	(missing)	Medication missing
Medication Name	Cetirizine	(missing)	Medication missing
Medication Name	Pantoprazole	(missing)	Medication missing
Medication Name	Levetiracetam	(missing)	Medication missing
Medication Name	Atorvastatin	(missing)	Medication missing
Medication Name	Perindopril	(missing)	Medication missing
Medication Name	Hydrocortisone / clotrimazole	(missing)	Medication missing
Medication Name	Pregabalin	(missing)	Medication missing
Medication Name	Lercanidipine	Unknown blood pressure medicine	Medication missing
Medication Name	Ferrous fumerate / folic acid	(missing)	Medication missing
Medication Name	Clonidine	(missing)	Medication missing
Medication Name	Epoetin lamda	epoetin	Medication missing
Medication Name	Mirtazapine	(missing)	Medication missing
Medication Name	Melatonin	(missing)	Medication missing
Medication Name	Hydrocortisone	(missing)	Medication missing
Medication Name	levetiracetam	(missing)	Medication missing
Medication Name	rivaroxaban	(missing)	Medication missing
Medication Name	venlafaxine	(missing)	Medication missing
Medication Name	Epoetin lamda	Epoetin	Medication missing
Medication Name	Amoxicillin / clavulanic acid	(unspecified antibiotic)	Medication missing
Medication Name	Carvediolol	(missing)	Medication missing
Medication Name	Lercanidipine	(missing)	Medication missing
Medication Name	Mometasone furoate	Unspecified topical cream	Medication missing
Medication Name	Nasal spray (over the counter)	(missing)	Medication missing
Medication Name	Hydrocortisone	(missing)	Medication missing
Medication Name	Amoxicillin / clavulanic acid	(missing)	Medication missing
Medication Name	Cetirizine	(missing)	Medication missing
Medication Name	Metformin	(missing)	Medication missing
Medication Name	Dapagliflozin	(missing)	Medication missing
Medication Name	Linagliptin	(missing)	Medication missing
Medication Name	Rosuvastatin	(missing)	Medication missing
Medication Name	Athletes foot cream, brand / drug unknown	(missing)	Medication missing
Medication Name	Prednisolone	(missing)	Medication missing
Medication Name	Pregabalin	(missing)	Medication missing
Medication Name	Fluconazole	Antifungal (generic name not specified)	Medication missing

Medication Name	Enoxaparin	Injection (generic name not specified)	Medication missing
Medication Name	Amoxicillin suspension	Oral liquid (generic name not specified)	Medication missing
Medication Name	Bisoprolol	(missing)	Medication missing
Patient Details	Julie Bell	Julie	Patient details missing
Patient Details	Prawns	Nil known	Patient details missing
Patient Details	Nil known	(missing)	Patient details missing
Patient Details	Lesley smart	Leslie	Patient details missing
Strength & Form	100 mg tablets	(missing) tablets	Strength or form missing
Strength & Form	2 mg modified release tablets	2 mg tablets	Strength or form missing
Strength & Form	11.6 mg/g gel	(missing) gel	Strength or form missing
Strength & Form	3.125 mg tablets	(missing) tablets	Strength or form missing
Strength & Form	100 mg / 25 mg tablets	(missing) tablets	Strength or form missing
Strength & Form	0.375 mg slow release tablets	(missing) slow-release tablets	Strength or form missing
Strength & Form	25 mg capsules	25 mg	Strength or form missing
Strength & Form	150mg slow release capsules	150 mg slow release	Strength or form missing
Strength & Form	40 mg tablets	(missing) tablet	Strength or form missing
Strength & Form	60 mg slow release tablets	60 mg tablets	Strength or form missing
Strength & Form	40 mg / 12.5 mg tablets	(missing)	Strength or form missing
Strength & Form	25,000 unit capsules	(missing) capsule	Strength or form missing
Strength & Form	8 mg oral disintegrating tablets	(missing) tablet	Strength or form missing
Strength & Form	200 mg tablets	tablet	Strength or form missing
Strength & Form	10 mg tablets	10 mg (missing form)	Strength or form missing
Strength & Form	100 mg injection	injection	Strength or form missing
Strength & Form	875 mg / 125 mg tablets	(missing)	Strength or form missing
Strength & Form	10 mg tablets	(missing) tablet	Strength or form missing
Strength & Form	2.5mg tablets	(missing) tablet	Strength or form missing
Strength & Form	250 mg tablets	(missing)	Strength or form missing
Strength & Form	5 mg tablets	5 mg (form not specified)	Strength or form missing
Strength & Form	75 mg tablets	75 mg (form not specified)	Strength or form missing
Strength & Form	1000 mg slow release tablets	(strength missing) extended-release tablets	Strength or form missing
Strength & Form	100 microg tablets	(missing) tablets	Strength or form missing
Strength & Form	40 mg tablets	(missing) tablets	Strength or form missing
Strength & Form	32 mg tablets	32 mg	Strength or form missing
Strength & Form	400 mg tablets	400 mg	Strength or form missing
Strength & Form	10 mg tablets	10 mg	Strength or form missing
Strength & Form	400 microg slow release tablet	(missing) strength slow-release preparation	Strength or form missing
Strength & Form	310 mg - 350 microg tablets	(missing) strength (form missing)	Strength or form missing
Strength & Form	10,000 unit / injection	(missing) strength injection	Strength or form missing
Strength & Form	5 mg tablets	(missing) tablets	Strength or form missing
Strength & Form	8 mg tablets	(missing)	Strength or form missing
Strength & Form	5 mg tablets	(missing)	Strength or form missing
Strength & Form	400 microg slow release tablets	(missing)	Strength or form missing
Strength & Form	3.125 mg tablets	(missing)	Strength or form missing

Strength & Form	850 mg tablets	(missing)	Strength or form missing
Strength & Form	100 mg tablets	(missing)	Strength or form missing
Strength & Form	10 mg tablets	(missing)	Strength or form missing
Strength & Form	25 mg capsules	(missing) capsule	Strength or form missing
Strength & Form	100 mg tablets	(missing) tablet	Strength or form missing
Strength & Form	875 mg / 125 mg	tablets (strength not stated)	Strength or form missing
Strength & Form	200 mg tablet	(missing)	Strength or form missing
Strength & Form	5 mg tablets	(missing)	Strength or form missing
Strength & Form	120 mg injection	injection (strength not stated)	Strength or form missing
Strength & Form	20 mg tablets	(missing)	Strength or form missing
Strength & Form	40 mg tablets	(missing)	Strength or form missing
Strength & Form	250 mg / 5 mL	(strength missing) syrup	Strength or form missing
Strength & Form	250 mg / 25 mg	(strength and form missing)	Strength or form missing
Strength & Form	1 mg / g cream	(strength missing) cream	Strength or form missing
Strength & Form	3.5 g sachet (or sachet)	(strength missing) sachet	Strength or form missing
Strength & Form	50 mg / 8 mg tablet	(strength and form missing)	Strength or form missing
Strength & Form	10 mg tablets	(strength not specified) tablets	Strength or form missing
Strength & Form	90 mg tablets	(strength not specified) tablets	Strength or form missing
Strength & Form	60 mg slow release tablet	(strength not specified) tablets	Strength or form missing
Strength & Form	500 mg tablets	(missing)	Strength or form missing
Strength & Form	40 mg tablets	(missing)	Strength or form missing
Strength & Form	60 mg injection	(missing) injection	Strength or form missing
Strength & Form	100 mg tablets	(missing)	Strength or form missing
Strength & Form	8 mg odt tablets	(missing)	Strength or form missing
Strength & Form	20 mg tablets	(missing) tablets	Strength or form missing
Strength & Form	25 mg tablets	(missing) tablets	Strength or form missing
Strength & Form	25,000 unit capsule	(missing) tablets	Strength or form missing
Strength & Form	240 mg Slow release tablets	(missing) tablet	Strength or form missing
Strength & Form	20 mg tablets	(missing) tablet	Strength or form missing
Strength & Form	32 mg tablet	(missing) tablet	Strength or form missing
Strength & Form	10 mg tablets	(missing) tablet	Strength or form missing
Strength & Form	75 mg capsules	(missing)	Strength or form missing
Strength & Form	60 mg capsules	(missing)	Strength or form missing
Strength & Form	10 mg tablets	(missing)	Strength or form missing
Strength & Form	1000 mg tablets	(missing) tablets	Strength or form missing
Strength & Form	40 mg tablets	(missing) tablets	Strength or form missing
Strength & Form	80 mg tablets	tablet	Strength or form missing
Strength & Form	5 mg tablets	(missing)	Strength or form missing
Strength & Form	2 mg tablets	(missing)	Strength or form missing
Strength & Form	10 mg tablets	(missing)	Strength or form missing
Strength & Form	15 mg tablets	(missing)	Strength or form missing
Strength & Form	5 mg tablets	(missing)	Strength or form missing
Strength & Form	10 mg tablets	(not stated)	Strength or form missing
Strength & Form	150 mg slow release capsule	(strength not specified) (form not specified)	Strength or form missing

Strength & Form	2 mg slow release tablets	Tablets (strength not specified)	Strength or form missing
Strength & Form	45 mg odt tablets	(missing) tablets	Strength or form missing
Strength & Form	500 microg / 400 microg slow release capsules	(missing) tablets	Strength or form missing
Strength & Form	1% / 1% cream	(missing) cream	Strength or form missing
Strength & Form	5 mg tablets	(unknown strength) tablet	Strength or form missing
Strength & Form	1000 mg slow release tablets	(unknown strength) tablet	Strength or form missing
Strength & Form	10 mg tablets	(unknown strength) tablet	Strength or form missing
Strength & Form	11.6 mg/g gel	(unknown strength) gel	Strength or form missing
Strength & Form	500 mg tablets	tablet (strength not stated)	Strength or form missing
Strength & Form	20 mg tablets	tablet (strength not stated)	Strength or form missing
Strength & Form	40 mg / 12.5 mg tablets	tablet (strength not stated)	Strength or form missing
Strength & Form	10 mg tablets	tablet (strength not stated)	Strength or form missing
Strength & Form	2 mg tablets	tablets, strength not specified	Strength or form missing
Strength & Form	0.25 microg capsules	tablets, strength not specified	Strength or form missing
Strength & Form	6000 unit injection	injection, strength not specified	Strength or form missing
Strength & Form	20 mg tablets	tablets, strength not specified	Strength or form missing
Strength & Form	5 mg tablets	(missing) tablets	Strength or form missing
Strength & Form	875 mg / 125 mg tablets	(missing) tablets	Strength or form missing
Strength & Form	120 mg injections	(missing) injection	Strength or form missing
Strength & Form	5 mg tablets	(missing) tablet	Strength or form missing
Strength & Form	50mg tablets	(missing) tablet	Strength or form missing
Strength & Form	100 microg tablets	(missing)	Strength or form missing
Strength & Form	25,000 unit capsules	(missing)	Strength or form missing
Strength & Form	1000 mg slow release tablets	(strength missing) tablet	Strength or form missing
Strength & Form	100 mg tablets	(strength missing) tablet	Strength or form missing
Strength & Form	2.5 mg tablets	(strength missing) tablet	Strength or form missing
Strength & Form	5 mg tablet	(strength missing) tablet	Strength or form missing
Strength & Form	60 mg slow release tablets	(missing)	Strength or form missing
Strength & Form	20 mg tablets	(missing)	Strength or form missing
Strength & Form	40 mg tablets	(missing)	Strength or form missing
Strength & Form	100 mg tablets	(missing)	Strength or form missing
Strength & Form	25 mg tablet	(missing)	Strength or form missing
Strength & Form	850 mg tablets	(missing)	Strength or form missing
Strength & Form	150 mg slow release capsules	Unknown strength tablets	Strength or form missing
Strength & Form	25 mg capsules	Unknown strength capsules	Strength or form missing
Strength & Form	500 mg tablets	Unknown strength tablets	Strength or form missing
Strength & Form	75 mg tablets	(missing)	Strength or form missing
Strength & Form	2.5 mg tablets	(missing)	Strength or form missing
Strength & Form	40 mg tablets	(missing)	Strength or form missing
Strength & Form	15 mg odt tablets	(missing) tablets	Strength or form missing
Strength & Form	100 mg / 25 mg tablets	(missing) tablets	Strength or form missing
Strength & Form	50 mg / 8 mg tablets	(missing) tablets	Strength or form missing
Strength & Form	3.5g sachet / powder	(missing) sachet	Strength or form missing
Strength & Form	15 mg tablets	(missing)	Strength or form missing

Strength & Form	20 mg tablets	(missing)	Strength or form missing
Strength & Form	5 mg tablets	(missing)	Strength or form missing
Strength & Form	875 mg / 125 mg tablets	(missing)	Strength or form missing
Strength & Form	5 mg tablets	(missing) tablets	Strength or form missing
Strength & Form	3.125 mg tablets	(missing)	Strength or form missing
Strength & Form	10 mg tablets	(missing)	Strength or form missing
Strength & Form	200 mg tablets	tablets (strength not specified)	Strength or form missing
Strength & Form	40 mg tablets	(missing)	Strength or form missing
Strength & Form	1000 mg tablets	(missing)	Strength or form missing
Strength & Form	145 mg tablets	(missing)	Strength or form missing
Strength & Form	80 mg tablets	(missing)	Strength or form missing
Strength & Form	8 mg tablets	(missing) tablet	Strength or form missing
Strength & Form	10 mg tablets	(missing)	Strength or form missing
Strength & Form	25 mg tablets	(missing) tablets	Strength or form missing
Strength & Form	1 % / 1 % cream	(missing)	Strength or form missing
Strength & Form	25 mg modified release tablets	(missing)	Strength or form missing
Strength & Form	60 mg capsules	(missing)	Strength or form missing
Strength & Form	5 mg tablets	(missing)	Strength or form missing
Strength & Form	100 mg tablets	(missing)	Strength or form missing
Strength & Form	75 mg capsules	(missing)	Strength or form missing
Strength & Form	37.5 mg slow release capsules	(missing)	Strength or form missing
Strength & Form	20 mg tablets	Unknown strength tablet	Strength or form missing
Strength & Form	310 mg / 310 microg tablets	(strength missing) (form missing)	Strength or form missing
Strength & Form	100 microg tablets	(strength missing) (form missing)	Strength or form missing
Strength & Form	2 mg tablets	(strength missing) tablet	Strength or form missing
Strength & Form	10,000 unit injections	(strength missing) injection	Strength or form missing
Strength & Form	1 mg tablets	(missing)	Strength or form missing
Strength & Form	40 mg tablet	(missing)	Strength or form missing
Strength & Form	45 mg odt tablets	(missing)	Strength or form missing
Strength & Form	45 mg odt tablet	(missing) tablet	Strength or form missing
Strength & Form	2 mg slow release tablet	(missing) tablet	Strength or form missing
Strength & Form	4 mg tablets	(missing) tablet	Strength or form missing
Strength & Form	100 mg tablet	(missing) tablet	Strength or form missing
Strength & Form	37.5 mg slow release capsules	(missing) tablet	Strength or form missing
Strength & Form	1000 mg tablets	(missing)	Strength or form missing
Strength & Form	15mg tablets	(missing)	Strength or form missing
Strength & Form	150 mg modified release capsules	(missing)	Strength or form missing
Strength & Form	10 mg tablets	(missing)	Strength or form missing
Strength & Form	145 mg tablets	(missing)	Strength or form missing
Strength & Form	2 mg tablets	(missing)	Strength or form missing
Strength & Form	1000 mg tablets	1 gram	Strength or form missing
Strength & Form	40 mg tablets	40 mg	Strength or form missing
Strength & Form	2 mg slow release tablets	(missing) tablet	Strength or form missing
Strength & Form	40 mg / 12.5 mg tablets	(missing)	Strength or form missing

Strength & Form	25 mg slow release tablets	(missing)	Strength or form missing
Strength & Form	50 mg / 8 mg tablets	(missing)	Strength or form missing
Strength & Form	3.5 g sachet	(missing) sachet	Strength or form missing
Strength & Form	6,000 unit injection	(missing strength) injection	Strength or form missing
Strength & Form	310 mg / 350 microg	(missing strength) tablet	Strength or form missing
Strength & Form	10 mg tablets	(missing strength) tablet	Strength or form missing
Strength & Form	150 mg slow release capsules	(strength missing) capsule	Strength or form missing
Strength & Form	20 mg tablets	(missing)	Strength or form missing
Strength & Form	60 mg injections	(missing) injection	Strength or form missing
Strength & Form	20 mg tablets	(strength missing) tablet	Strength or form missing
Strength & Form	50 mg tablets	(strength missing) tablet	Strength or form missing
Strength & Form	0.1% cream	(strength missing) cream	Strength or form missing
Strength & Form	Missing	(strength missing) tablet	Strength or form missing
Strength & Form	5mg tablets	(missing)	Strength or form missing
Strength & Form	5 mg tablets	(missing)	Strength or form missing
Strength & Form	60 mg slow release tablets	(missing)	Strength or form missing
Strength & Form	40 mg tablets	(missing)	Strength or form missing
Strength & Form	2.5 mg tablets	(missing)	Strength or form missing
Strength & Form	100 mg tablets	(missing)	Strength or form missing
Strength & Form	10 mg tablet	(missing)	Strength or form missing
Strength & Form	10 mg tablets	(missing)	Strength or form missing
Strength & Form	850 mg tablets	(missing)	Strength or form missing
Strength & Form	40 mg tablets	(missing)	Strength or form missing
Strength & Form	5 mg tablet	(missing)	Strength or form missing
Strength & Form	80 mg tablets	(missing) tablets	Strength or form missing
Strength & Form	100 mg tablets	(missing) tablets	Strength or form missing
Strength & Form	20 mg tablets	(missing) tablets	Strength or form missing
Strength & Form	750 mg tablets	strength not specified tablets	Strength or form missing
Strength & Form	10 mg tablets	strength not specified tablets	Strength or form missing
Strength & Form	25 mg capsules	(missing)	Strength or form missing
Strength & Form	25 mg tablets	(missing)	Strength or form missing
Strength & Form	3.125 mg tablet	strength not specified (form missing)	Strength or form missing
Strength & Form	24 mg / 26 mg tablets	strength not specified capsule	Strength or form missing
Strength & Form	40 mg tablets	strength not specified (form missing)	Strength or form missing
Strength & Form	10 mg tablets	(missing) tablets	Strength or form missing
Strength & Form	20 mg tablets	(missing) tablets	Strength or form missing
Strength & Form	400 mg tablets	(missing) tablets	Strength or form missing
Strength & Form	100 microg tablets	(missing) tablets	Strength or form missing
Strength & Form	250 mg tablet	(missing) (missing form)	Strength or form missing
Strength & Form	The smallest one	(missing) capsules	Strength or form missing
Strength & Form	120 mg injections	(strength missing) (form missing)	Strength or form missing
Strength & Form	200 mg capsules	(strength missing) capsules	Strength or form missing
Strength & Form	875 mg / 125 mg tablets	(strength missing) (form missing)	Strength or form missing
Strength & Form	1 g (missing)	(strength missing) (form missing)	Strength or form missing

Strength & Form	20 mg tablet	(missing) tablets	Strength or form missing
Strength & Form	40 mg tablet	40 mg (missing)	Strength or form missing
Strength & Form	145 mg tablet	(missing) tablets	Strength or form missing
Strength & Form	37.5 mg slow release capsule	(missing)	Strength or form missing
Strength & Form	32 mg tablet	(missing)	Strength or form missing
Strength & Form	2.5 mg tablet	(missing)	Strength or form missing
Strength & Form	100 mg tablet	(missing)	Strength or form missing
Strength & Form	5 mg tablet	(missing)	Strength or form missing
Strength & Form	150 mg slow release capsules	(missing)	Strength or form missing
Strength & Form	2 mg modified release tablets	(missing)	Strength or form missing
Strength & Form	15 mg odt tablet	(missing) tablet	Strength or form missing
Strength & Form	100 microg tablet	(missing) tablet	Strength or form missing
Strength & Form	20 mg tablets	(missing) tablets	Strength or form missing
Strength & Form	3.125 mg tablets	(missing)	Strength or form missing
Strength & Form	310 mg / 350 microg tablets	tablets	Strength or form missing
Strength & Form	10,000 unit injections	injection	Strength or form missing
Strength & Form	25 mg tablets	(strength and form missing)	Strength or form missing
Strength & Form	310 mg / 350 microg	(strength missing) tablets	Strength or form missing
Strength & Form	3.5 g sachet	(strength missing) sachets	Strength or form missing
Strength & Form	25 mg slow release tablets	(missing) tablets	Strength or form missing
Strength & Form	32 mg tablets	(missing) tablets	Strength or form missing
Strength & Form	50mg / 8mg tablets	(missing) tablets	Strength or form missing
Strength & Form	40 mg tablerts	(strength not specified) tablet	Strength or form missing
Strength & Form	2 mg tablets	(strength not specified) tablet	Strength or form missing
Strength & Form	60 mg slow release tablets	60 mg tablet	Strength or form missing
Strength & Form	50 mg tablets	50 mg (form missing)	Strength or form missing
Strength & Form	0.1% cream	cream (strength missing)	Strength or form missing
Strength & Form	20 mg tablets	20 mg (form missing)	Strength or form missing
Strength & Form	probably 250 microg	inhaler (strength missing)	Strength or form missing
Strength & Form	24 mg / 26 mg	(missing strength) tablet	Strength or form missing
Strength & Form	5 mg tablets	(missing strength) tablet	Strength or form missing
Strength & Form	4 mg tablets	(missing strength) tablet	Strength or form missing
Strength & Form	25 mg capsules	(missing strength) capsule	Strength or form missing
Strength & Form	20 mg tablets	(missing strength) (missing form)	Strength or form missing
Strength & Form	875 mg / 125 mg tablets	(strength missing) tablets	Strength or form missing
Strength & Form	10 mg tablets	(strength missing) tablets	Strength or form missing
Strength & Form	1000 mg slow release tablets	(missing)	Strength or form missing
Strength & Form	10 mg tablets	(missing)	Strength or form missing
Strength & Form	5 mg tablet	(missing)	Strength or form missing
Strength & Form	25 mg tablet	(missing)	Strength or form missing
Strength & Form	1000 mg slow release tablet	(missing) tablet	Strength or form missing
Strength & Form	80 mg tablets	(missing) tablet	Strength or form missing
Strength & Form	40 mg tablets	(missing) tablets	Strength or form missing
Strength & Form	8 mg tablets	(missing) tablets	Strength or form missing

Strength & Form	5 mg tablets	(missing) tablets	Strength or form missing
Strength & Form	1 mg tablets	(missing) tablets	Strength or form missing
Strength & Form	5 mg tablets	(missing) tablets	Strength or form missing
Strength & Form	75 mg capsules	(missing) tablets	Strength or form missing
Strength & Form	200 mg capsules	Capsule (strength not specified)	Strength or form missing
Strength & Form	60 mg injection	Injection (strength not specified)	Strength or form missing
Strength & Form	250 mg in 5 mL	Liquid (strength not specified)	Strength or form missing
Strength & Form	25,000 unit capsules	Capsules (strength not specified)	Strength or form missing
Strength & Form	2.5 mg tablet	(missing)	Strength or form missing
Strength & Form	10 mg tablets	(missing)	Strength or form missing
Strength & Form	5 mg tablets	(missing)	Strength or form missing
